# Research progress on maintaining chloroplast homeostasis under stress conditions: a review: Chloroplast homeostasis under stress conditions

**DOI:** 10.3724/abbs.2023022

**Published:** 2023-02-24

**Authors:** Qi Wang, Jiang Yue, Jianmin Yan

**Affiliations:** 1 College of Agriculture Guizhou University Guiyang 550025 China; 2 Vegetable Research Academy Guizhou University Guiyang 550000 China

**Keywords:** environmental stress, chloroplast quality control, protein turnover, TOC-TIC, retrograde signal, chloroplast degradation

## Abstract

On a global scale, drought, salinity, extreme temperature, and other abiotic stressors severely limit the quality and yield of crops. Therefore, it is crucial to clarify the adaptation strategies of plants to harsh environments. Chloroplasts are important environmental sensors in plant cells. For plants to thrive in different habitats, chloroplast homeostasis must be strictly regulated, which is necessary to maintain efficient plant photosynthesis and other metabolic reactions under stressful environments. To maintain normal chloroplast physiology, two important biological processes are needed: the import and degradation of chloroplast proteins. The orderly import of chloroplast proteins and the timely degradation of damaged chloroplast components play a key role in adapting plants to their environment. In this review, we briefly describe the mechanism of chloroplast TOC-TIC protein transport. The importance and recent progress of chloroplast protein turnover, retrograde signaling, and chloroplast protein degradation under stress are summarized. Furthermore, the potential of targeted regulation of chloroplast homeostasis is emphasized to improve plant adaptation to environmental stresses.

## Introduction

As the global environment deteriorates, crops in many parts of the world are vulnerable to various stressors. The deterioration caused by drought, temperature, and erosion, as well as increased soil salinity due to excessive fertilization or irrigation patterns, has significantly affected crop growth [1]. Furthermore, prime agricultural land is shrinking in many areas due to population growth and urban expansion, seriously affecting the yield and agronomic traits of crops [2]. Therefore, improving the environmental adaptability of plants is of great significance to the sustainable development of agriculture and the environment. The electron transport chain in plant chloroplasts allows the reception of energy from sunlight. In harsh environments, the photosynthesis of plants is inhibited, and the ability of chloroplasts to transfer electrons exceeds the threshold required by plants. As a result, excessive energy accumulation occurs, producing reactive oxygen species (ROS) [3]. ROS cause rapid oxidative damage to DNA, proteins, lipids, and other cellular components. In addition to the primary function of photosynthesis, plant chloroplasts can sense environmental stress and respond rapidly to stress. It is widely believed that chloroplasts originate from prokaryotic cyanobacteria [4]. Since the genetic material of chloroplasts was transferred from the prokaryotic genome to the eukaryotic nucleus during evolution, most chloroplast proteins are encoded by the nucleus. Although the chloroplast has its genome, only approximately 100 chloroplast proteins are encoded by the chloroplast genome [ 5, 6] . Approximately 95% (approximately 2000‒2500) of chloroplast proteins are encoded by the nucleus. These proteins exist in the cytoplasm as precursor proteins and are transported to the chloroplast through the complex of the translocons at the outer envelope membrane of chloroplasts (TOC) and the translocons at the inner envelope membrane of chloroplasts (TIC), named TOC-TIC, on the chloroplast envelope. By providing the material and functional basis for chloroplast development, these proteins ensure the proper function of the chloroplast.

Correct transport and assembly of the chloroplast proteome are necessary for normal plant function, plastid development, and physiological events. However, plants can be induced to produce ROS under environmental stress, damaging chloroplast membrane components and even destroying entire chloroplasts. Damage to the chloroplast membrane impairs the translocation of important nuclear genome-encoded proteins by TOC-TIC complexes on the membrane [7]. The turnover process of chloroplast proteins is disturbed, inhibiting normal growth and developmental processes in crops, including photosynthesis, mRNA modification, transcription, protein synthesis, amino acid synthesis, and lipid metabolism [8]. To address these problems, plants have evolved adaptive mechanisms to maintain proteostasis in the chloroplast under stress, including retrograde signaling, changes in gene expression or cell physiology, and other posttranslational mechanisms [ 9– 11] . Normal turnover and homeostasis in chloroplasts are crucial for maintaining normal plant growth under stress conditions. Understanding the physiological and molecular mechanisms of chloroplast homeostasis under stress conditions is essential for improving crop stress resistance and crop-directed improvement. In recent years, many breakthroughs have been made in this field.

In this review, we describe the regulatory mechanism of chloroplast homeostasis under stress conditions, which involves chloroplast protein turnover, retrograde signal and chloroplast degradation.

## TOC-TIC Protein Import Apparatus

TOC and TIC together mediate the transport of precursor proteins across the chloroplast envelope [12]. Although many mechanistic details of the TOC-TIC complex transport system remain to be defined, researchers have reached a consensus on the core components and functions of the TOC complex. The core components of the TOC complex include two GTPases (Toc159 and Toc34) and the outer membrane transport channel (Toc75) [13]. Nuclear-encoded precursor proteins contain cleavable transit peptides (TPs) at the N-terminus, and targeting signals in TPs guide them to the chloroplast surface [14]. During the targeting process, multiple chaperone proteins bind to precursor proteins to maintain the linear structure of the cytosolic protein. Subsequently, cytokines on the chloroplast surface and two GTPase receptors on the outer chloroplast membrane specifically recognize the targeting signal, and hydrolysed GTP facilitates the passage of precursor proteins through the Toc75 channel [ 15, 16] . Currently, the composition of the TIC apparatus is controversial. Initial studies showed that several proteins, including Tic20, Tic22, Tic21, Tic40, and Tic110, form the inner membrane protein import complex [ 17, 18] . However, few of these proposed TIC candidate components have been detected in early models. Recent studies have revised earlier TIC models by identifying a Tic20-centered 1 MDa complex [19] in *Arabidopsis*, which was demonstrated to be a TIC complex working in concert with the TOC apparatus. Furthermore, recent studies on *Chlamydomonas* have fully confirmed the presence of TIC 1 MDa complexes in green algae [20]. In addition to Tic20, the 1 MDa complex also contains 3 essential proteins, including Tic214, Tic100, and Tic56. Despite these findings, the detailed function of each subunit in the 1 MDa complex remains to be elucidated. Its cooperation with the TOC complex in transporting preproteins also needs to be investigated.

## The TOC Apparatus Regulates Chloroplast Protein Import under Stressful Conditions

For photooxidation caused by the energy overflow of photosynthesis in plants exposed to stress conditions, plants can mitigate the damage by different methods, such as nonphotochemical quenching, state transitions, and accumulation of ROS scavengers [ 21, 22] . These protective mechanisms are important in avoiding photooxidative damage to chloroplast proteins and maintaining chloroplast protein homeostasis. Although the photoprotective mechanisms in plants are important for resisting photooxidative damage, an introduction to photoprotection and an elucidation of the detailed molecular mechanisms are beyond the scope of this paper.

As a key starting point for protein import, the TOC-TIC protein import apparatus has received attention in regulating chloroplast homeostasis.

Under external stress conditions, chloroplast proteins are severely damaged by photooxidation, and the protein import apparatus on the membrane is also disrupted. External stress usually leads to increased degradation of chloroplast proteins and decreased synthesis of chloroplast proteins, resulting in changes in the protein turnover rate [23]. Therefore, plants maintain normal chloroplast protein transport by adjusting the level of protein import machinery, which is an important means to cope with oxidative damage under stress [ 24, 25] .

Under high-temperature stress, the expression of the TOC-TIC protein import apparatus was differentially downregulated, and the photosynthesis of plants was downregulated due to the reduced import of plastid proteins encoded by nuclear genes. The reduced protein import into chloroplasts alleviates the temperature stress-induced impairment of photooxidation and reactive oxygen species production in plants [26]. In addition, the net loss of photosynthesis and other plastid functions under high-temperature stresses may be due to the degradation of chloroplast proteins [27] and the impairment of posttranslational targeting of their precursor proteins in the chloroplast. To maintain normal chloroplast function, some newly synthesized proteins need to be imported into the chloroplast in large quantities to replenish the damaged proteins. However, no significant changes in the transcriptional level or protein abundance of Toc34 were observed under heat stress, which may be an adaptive mechanism for chloroplasts to maintain protein import efficiency. At extreme temperatures, molecular chaperones can also protect the TOC-TIC protein import apparatus from thermal denaturation [26]. It maintains the efficiency of protein transport and supports basic photosynthetic functions. Salt stress affects plant metabolism, such as photosynthesis and osmotic inhibition of root water uptake, slowing the growth rate of plants [ 28, 29] . Under salt stress, the expression of the Toc159 family in tomatoes is increased, contributing to the maintenance of basic plastid functions in plants under stress conditions and increasing the import of stress-related proteins [30]. Yan *et al*. [30] significantly promoted the growth of tomato plants under salt stress using rhizobacteria growth-promoting bacteria. The mechanism of action might be through the upregulation of the TOC GTPase genes (chloroplast protein import apparatus gene) by growth-promoting bacteria. Recent studies have revealed that under external stress conditions, SP1, a RING-type ubiquitin E3 ligase located in the plastid outer envelope membrane could control the level of specific TOC complex components, degrade components of the transport photosynthesis-related protein apparatus, reduce the import of photosynthesis-related proteins, and inhibit the excessive production of ROS under photooxidation [31]. Plants overexpressing the *SP1* gene exhibited greater abiotic stress tolerance than wild-type plants, while *SP1* mutants showed the opposite result [ 31, 32] . Regulation of protein import into peroxisomes and mitochondria may also contribute to the stress-tolerant phenotype in plants. These two organelles are also involved in ROS generation and plastid signaling [33], suggesting that regulating plastid protein transport can enhance plant stress tolerance.

**Figure 1:**
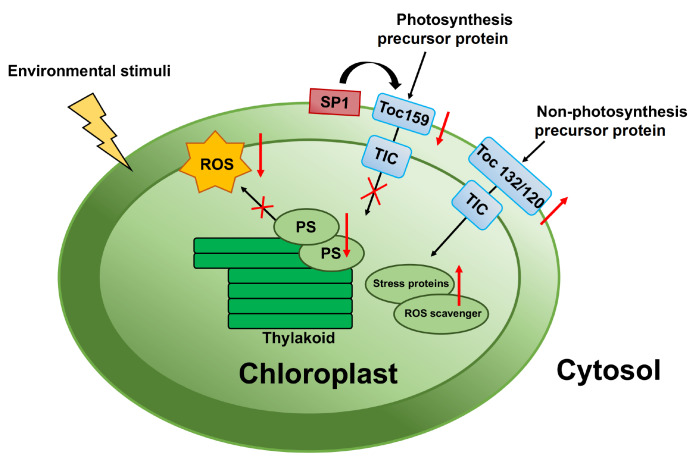
Figure 1 Regulation of the plastid general import machinery under stressful conditions Different TOC complexes mediate the import of specific classes (photosynthesis proteins and nonphotosynthesis proteins) of nuclear-encoded preproteins into plastids. The specificity of the import complexes is determined by Toc159 family receptors. Toc159 mainly transports photosynthesis-related proteins, and Toc132/Toc120 mainly transports nonphotosynthesis-related proteins. Under stress conditions, SP1 mediates a decrease in Toc159 abundance throughout the complex, reducing photosynthetic protein import to minimize photooxidative damage. Under less severe stress, Toc132/120 abundance is upregulated, promoting the substantial import of ROS scavengers, stress response, and other nonphotosynthetic proteins to defend against adversity.

To date, the TOC-TIC chloroplast protein import apparatus has rarely been studied in response to stress. However, the dynamic changes in the stoichiometry of each subunit of the TOC-TIC multisubunit protein transport apparatus are crucial for maintaining normal protein transport and repairing chloroplast function under stress. By adjusting the protein abundance of different subunits of the TOC complex, the import of photosynthesis-related proteins is reduced, avoiding photooxidative damage under stress. At the same time, the import of chloroplast proteomes associated with stress resistance is positively affected under stress, enabling plants to improve their adaptation to abiotic stresses ( Figure 1).

## Plastid-nuclear Retrograde Signaling Regulates Chloroplast Homeostasis

For good survivability, plants must be able to monitor their surroundings and make rapid adjustments in metabolic and physiological processes according to the severity of environmental stress [34]. Coordinated gene expression between the chloroplast and nuclear genomes of higher plants is critical for chloroplast biogenesis, normal chloroplast function, and maintenance of metabolic levels under stressful conditions [ 35, 36] . The regulation of nuclear gene expression by the chloroplast is achieved by retrograde signals from chloroplasts. Much evidence suggests that this signal regulates the development of the chloroplast and widely participates in regulating nuclear gene expression in response to adaptive regulation and stress in plants [37]. When genetic mutations or extreme environmental conditions affect chloroplast development, retrograde signaling reduces PhANG expression. Consequently, photosynthesis-related proteins are only expressed when the chloroplast is ready, thus avoiding photooxidative damage [38]. Four major pathways have been identified for retrograde signal regulation of nuclear transcription by chloroplasts: plastid gene expression (PGE); α-tocopherol-binding protein (TBP)-related signal; altered photosynthetic electron transport (PET) chain activity and its production of reactive oxygen species; and signaling molecules from impaired plastid metabolism [ 34, 39, 40] . Among them, the signals triggered by PGE and TBP are biogenic signals and mainly function in chloroplast biogenesis. In contrast, the metabolites and ROS produced by impaired plastid metabolism are operational signals. Their biological significance lies in regulating chloroplast and cellular homeostasis in response to external environmental stimuli [41].

**Figure 2:**
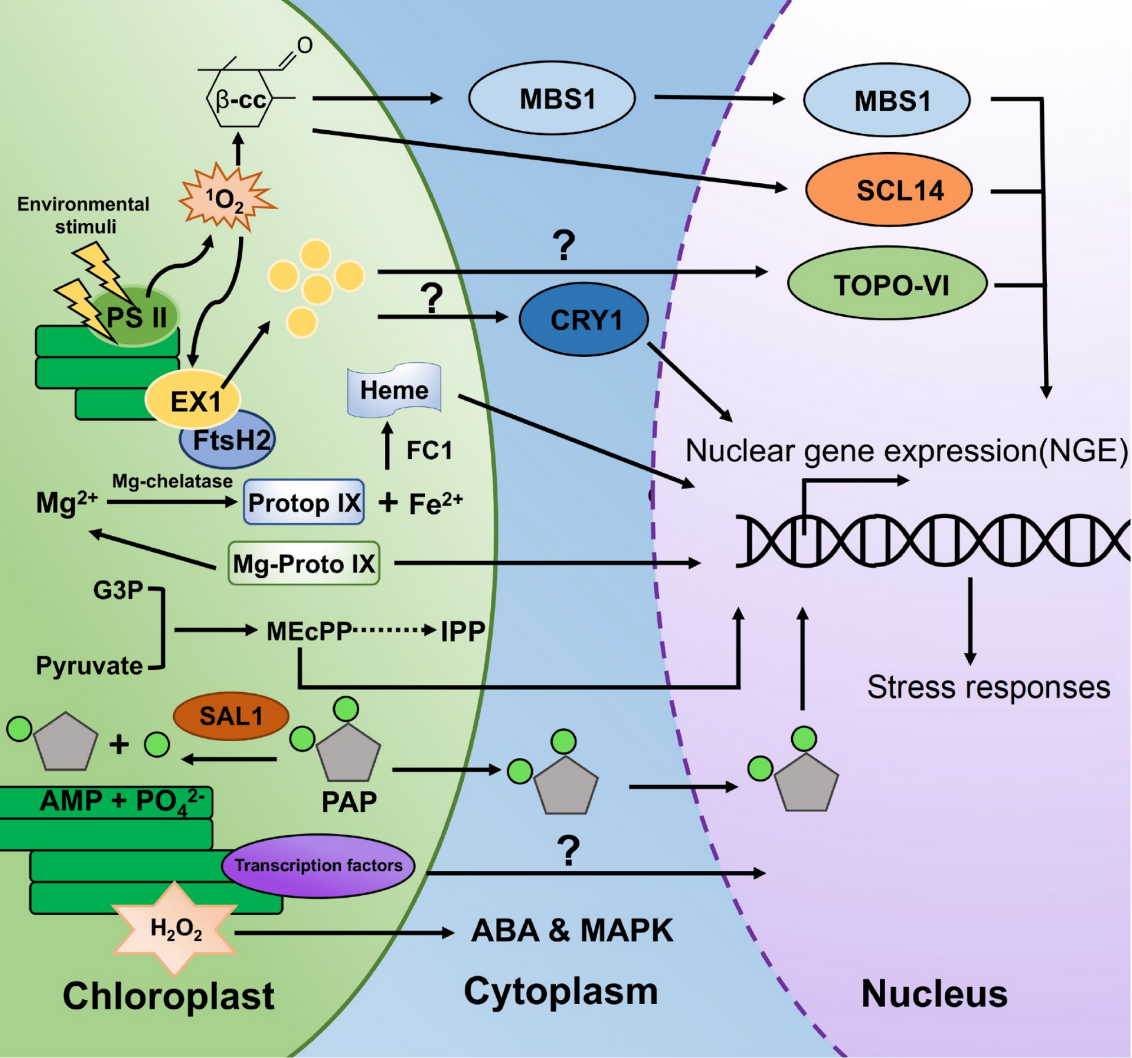
Figure 2 A schematic representation of a typical retrograde signaling pathway in plant cells Stress induces the accumulation of 1O 2, which leads to the accumulation of β-cyclic lemons (β-cc) in chloroplasts. β-Cyclic citrate is exported to the nucleus, where it signals to the nucleus via MBS1 and SCL14 to regulate the expressions of defense genes. 1O 2 causes proteolysis of the EXECUTOR 1 (EX1) protein via FtsH2 protease. Afterward, EX1 degradation products can signal to the nucleus through two mechanisms: modulation of cellular degradation by Cry1-dependent blue light signaling and topoisomerase VI (TOPO-VI). Both Mg-ProtoIX and heme in the tetrapyrrole pathway can be regulated by FC1, translocating from the chloroplast to the nucleus and regulating genes involved in photosynthesis. The methylsilanol 4-phosphate (MEP) pathway is also involved in the retrograde signaling pathway, and high light also induces the production of methylerythritol cyclic phosphate (MEcPP) in chloroplasts, which then regulates nuclear gene expression. 3′-Phosphate adenosine 5′-phosphate (PAP) induced by drought and high light can translocate from chloroplasts to the nucleus and regulate the expressions of a set of genes. H 2O 2 regulates mitogen-activated protein kinase-like enzyme (MAPK) in Arabidopsis and cooperates with ABA.

Considerable progress has been made in the study of chloroplast retrograde signaling. In terms of the stress response, several operational retrograde signals have been identified ( Table 1), including ROS [42], tetrapyrroles Mg-ProtoPIX [43], carotenoid oxidation products [44], phosphoadenosine [45], isoprenoid precursor methylerythritol cyclodiphosphate (MEcPP) [46], transcription factors [47], and kinases [48]. These seemingly unrelated component pathways form a complex network regulating diverse processes. Although many operational signaling mechanisms and intrinsic interactions remain unclear, they contribute to the regulation of nuclear gene expression involved in photosynthesis and stress adaptation, thereby modulating photochemical plant responses and reducing oxidative damage ( Figure 2).

**Table:** **Table 1** Source of typical retrograde signals in plants

Group	Typical member	Source	Ref.
ROS	^1^O _2_	Photooxidative damage products	[49]
	H _2_O _2_		[ 50, 51]
	O _2_^·–^		[ 52, 53]
Tetrapyrrole	Mg-ProtoIX	Chlorophyll biosynthetic intermediates	[54]
	heme	The heme branch of the plastid tetrapyrrole biosynthesis pathway	[55]
	bilins	Heme-derived linear tetrapyrroles	[56]
β-Carotene	β-cyclocitral	Carotenoid oxidation products	[44]
Sulfation	PAP	Product of PAPS sulfation reaction catalyzed by cytoplasmic sulfotransferase	[45]
Methylerythritol isoprenoid	MEcPP	A precursor of isoprenoids produced by the plastidial methylerythritol phosphate (MEP) pathway	[46]
Transcription factor	AP2	The family of Apetala 2 (AP2)/ethylene response element binding protein (EREBP) transcription factors	[47]
	Whirly1	Single-stranded DNA-binding protein family member	[ 57, 58]
	ABI4	Master AP2-type transcription factor	[59]
Kinase	MAPK6	Mitogen-activated protein kinase	[48]

## Multiple Degradation Pathways of Chloroplast Proteins under Stressful Conditions

**Figure 3:**
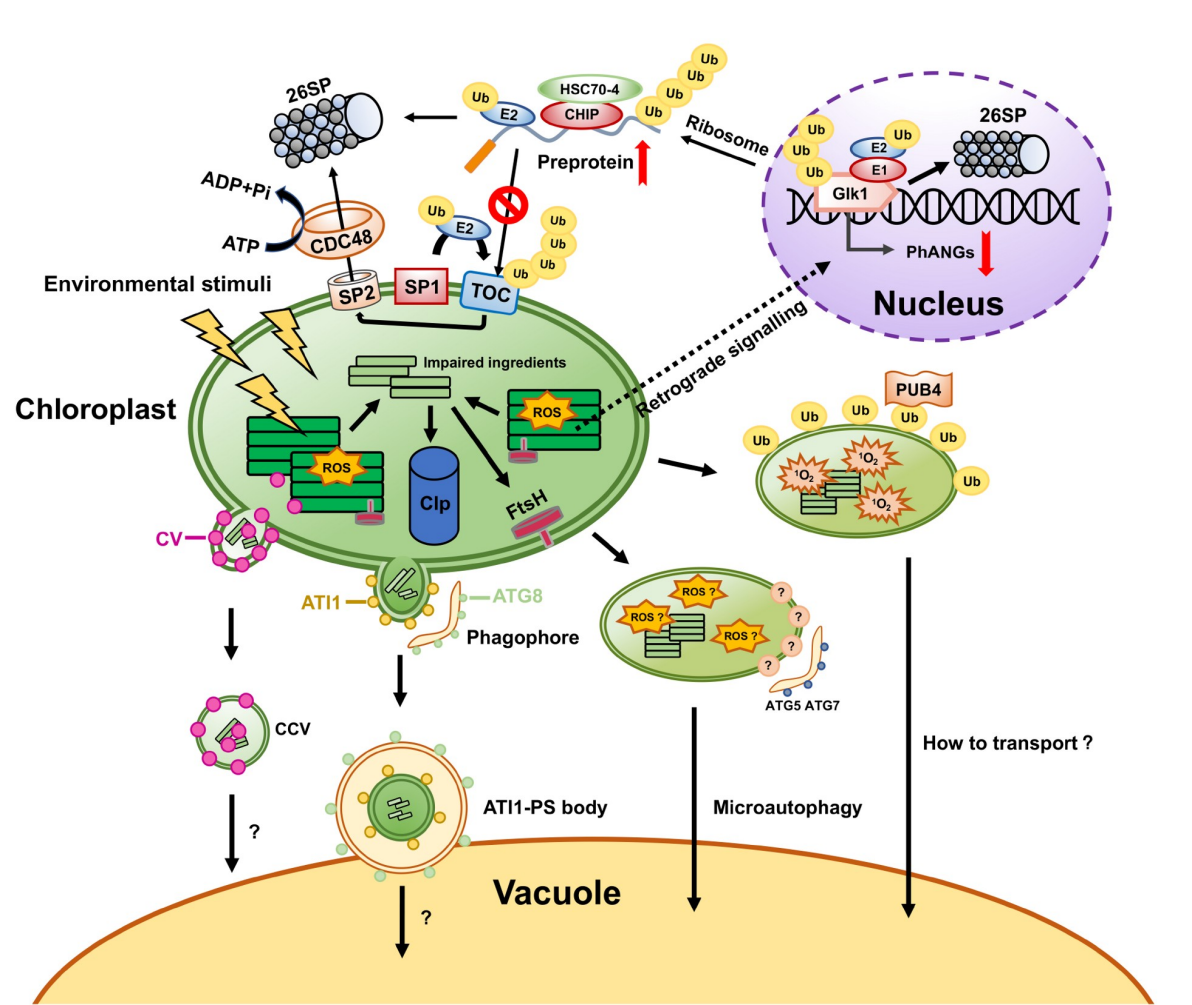
Figure 3 Schematic diagram of the chloroplast degradation pathway under stress conditions Chloroplast protease (Clp, FstH) degrades the damaged components. At stress or specific developmental stages, the UPS system directly targets the TOC apparatus for proteolysis, which is mediated by the E3 ligase SP1. The ubiquitin-labelled TOC protein is inverted from the membrane through the channel protein SP2 and powered by the cytoplasmic AAA+ ATPase CDC48 for degradation through the 26S proteasome. The excess accumulated precursor protein is degraded in the cytoplasm by the CHIP/HSC70-4 E3 ubiquitin ligase system. Golden-like 1 (GlK1) is a transcription factor that regulates the expression of nuclear-encoded phANGs. It may be degraded by the UPS in response to unknown retrograde signals induced by developmental processes or stress. The accumulation of 1O 2 in damaged chloroplasts activates chloroplast ubiquitination mediated by the cytosolic ubiquitin E3 ligase plant-box 4 (PUB4), and the mechanism by which ubiquitin-labelled chloroplasts are transported to the vacuole has not been clearly described. An autophagic process known as chlorophagy delivers damaged chloroplasts to the vacuole via microautophagic membrane kinetics requiring ATG5 and ATG7 functions. ATI1-PS (ATG8-Interacting PROTEIN 1) bodies are formed on chloroplasts, which encapsulate oxidized chloroplast components and are transported to vacuoles for degradation via ATG-mediated autophagy. The plastid transmembrane protein CV mediates the formation of CCV vesicles on the chloroplast surface, encases damaged components and is transported to the vacuole in an ATG-independent manner.

Under stress conditions, the excess energy produced in chloroplasts leads to sustained oxidative damage. Plant cells need to promptly remove and degrade chloroplasts damaged by ROS. Chloroplasts regulate degradation and maintain a healthy population of chloroplasts within the cell to support essential cellular functions and reduce photooxidative damage. In addition, nutrient recycling is important for plant survival under abiotic stresses. In leaves, 80% of nitrogen is found in chloroplasts [60], and degradation products from damaged chloroplasts allow the recycling and redistribution of these nutrients [61], which is a regulatory mechanism or plant adaptation to stressful environments. Four pathways of stress-induced chloroplast degradation are introduced in this review ( Figure 3), including the chloroplast endogenous protease system, the ubiquitin protease system (UPS), selective autophagy, and the chloroplast vesiculation (CV) pathway.

### Chloroplast endogenous protease system

Chloroplasts have a protease system for hydrolysing damaged or unwanted chloroplast components within the chloroplast to maintain internal protein homeostasis. A central component of the Clp chloroplast protease network [ 62, 63] , especially the ClpC chaperone, is involved in precursor protein transport and interacts with TIC [64]. The thylakoid FtsH protease degrades damaged PSII core protein and promotes the reassembly of photosystem II [ 65, 66] . Deg proteins function in photosystem II assembly and damage repair cycles [ 67, 68] .

### Ubiquitin protease system

The endogenous chloroplast protease system cannot regulate proteolysis outside the chloroplast matrix. Therefore, the cytoplasmic proteolytic pathway UPS needs to be involved.

UPS mediates the degradation of ubiquitinated proteins by the 26S protease complex in the cytoplasm. Ubiquitinated proteins from the chloroplast, mitochondrion, endoplasmic reticulum, and cytoplasm can be substrates for the 26S protease complex [69]. UPS has now been recognized as an important regulator of chloroplast quality [31]. Under stress conditions, the targeted import of precursor proteins into the chloroplast is disrupted, leading to the accumulation of precursor proteins in the cytoplasm with toxic effects on cells [70]. UPS can selectively degrade transcription factors expressed by photosynthesis-related chloroplast precursor proteins to reduce the accumulation of precursor proteins in the cytoplasm to form toxic aggregates. The *Arabidopsis* transcription factor GLK1 promotes the expression of photosynthetic proteins and has been identified as a key regulator of chloroplast biogenesis [71]. The posttranslational level of GLK1 can be regulated by UPS. At the plant seedling stage, selective degradation of the transcription factor GLK1 by UPS is triggered, reducing the expression of PhANGs at specific developmental stages. This may also be another mechanism for reducing photooxidation under stress. In addition to the previously described chloroplast retrograde signaling that reduces the expression of photosynthesis-related nuclear-encoded proteins, plant cells also degrade chloroplast precursor proteins accumulated in the cytoplasm through the UPS pathway. This degradation process was initially discovered in studies targeting E3 ubiquitin ligases [70]. The E3 ubiquitin ligase CHIP from *Arabidopsis* allowed *in vitro* ubiquitination of the FtsH protease complex subunit FtsH1 and was subsequently shown to direct the degradation of two chloroplast internal protease precursors (Clp and FtsH) under high light conditions [ 72, 73] . In the *Arabidopsis* Toc159 mutant ppi2, the chloroplast precursor protein is not efficiently imported due to the lack of the protein transporter, while the cytoplasmic chaperone Hsc70-4 is significantly upregulated in ppi2. By recruiting CHIP, accumulated precursor proteins are degraded by the proteasome [70]. Hsp70 and Hsp90 are also involved in the accumulation of chloroplast precursor proteins in tomatoes, modulating the abundance of plastid precursor proteins [74]. A previous study also identified the wheat E3 ubiquitin ligase stress-associated protein 5 (SAP5), which triggers the degradation of the Hsp90C precursor protein [75]. SP1 and SP2 are two newly discovered E3 ubiquitin ligases. As described above, SP1 regulates the protein abundance of the chloroplast membrane protein TOC complex for photochemical reactions under stressful conditions, with the mechanism of action mediated by SP1 [76]. The SP1-mediated UPS system directly targets the TOC apparatus for the ubiquitination of TOC proteins, which are subsequently degraded by the 26S proteasome. The whole process of SP2 and CDC48 is thought to be associated with the reverse translocation of ubiquitinated TOC [71]. The UPS degradation pathway includes the cooperation of SP1, SP2, and CDC48 proteins. This selective degradation pathway involving UPS for the TOC apparatus is also known as CHLORAD [76]. More importantly, the UPS mechanism also targets organelles [77]. Studies have revealed the role of ubiquitination in regulating the response of individual chloroplasts to environmental stress. Excessive amounts of protoporphyrin IX accumulate in the chloroplasts of *Arabidopsis* *fc1* and *fc2* mutants and produce singlet oxygen. Damaged chloroplasts are marked by ubiquitination, accelerating the UPS-mediated selective degradation of damaged chloroplasts [78]. Genetic screening demonstrated that the PUB4 E3 ubiquitin ligase is required for this process.

### Autophagy pathway

Autophagy is a conserved mechanism for transporting proteins, organelles, and other macromolecular substances to eukaryote vacuoles or lysosomes for degradation [79]. This system allows for massive degradation of intracellular protein and component collections, essential for the efficient recycling of cellular components to enable plants to adapt to biotic/abiotic stress, impaired oxidation, and nutrient starvation [80]. The autophagic process requires the formation of a double-membrane structure called a phagocyte to transport target degradants. Phagosome formation requires signal activation through a protein-conjugated cascade, which is controlled by a large number of autophagy-related proteins (ATGs) [81], a process similar to ubiquitin activation and the transfer of E1s, E2s, and E3s in the UPS [82]. Currently, chloroplasts have two autophagy-dependent degradation pathways: (i) piecemeal-type autophagy via Rubisco-containing bodies (RCBs), ATI1 (ATG8-INTERACTING PROTEIN1)-positive (ATI1-PS) bodies, and small starch-like granule (SSLG) bodies; and (ii) transport of whole chloroplasts to vacuoles (chlorophagy).

#### Piecemeal-type autophagy

The piecemeal chlorophagy pathway transports these chloroplast protein components into vacuoles for degradation, forming small sac-like structures that encapsulate Rubisco proteins, chloroplast matrix-targeting proteins, or small starch granules. These piecemeal autophagy pathways mediated by phagocytes are mainly involved in the process of nutrient reactivation for plant senescence and adaptation to carbon starvation [ 83, 84] . A detailed introduction of RCBs and SSLGs can be found in the literature [85]. ATI1 has been demonstrated to interact with many proteins involved in photosynthesis and the regulation of oxidative stress. Other studies revealed that the number of ATI1-PS bodies formed during salt stress increases, while ATI1 mutants show extreme intolerance to salt stress, demonstrating the effect of ATI1 in oxidative damage and removal of damaged chloroplast components under salt stress [86].

#### Chlorophagy

In addition to the degradation of some groups within the chloroplast, pressure-induced chloroplast oxidation damage can also cause vacuole degradation of the whole damaged chloroplast, a process known as chlorophagy [87]. This process is a selective autophagy process requiring the participation of ATG5, ATG7, and other core ATG proteins [ 87, 88] . It has been demonstrated that the response of NBR1 to insoluble ubiquitin whitening in *Arabidopsis* and tobacco mediates selective autophagy [ 89, 90] . The role of NBR1-mediated selective autophagy in the plant stress response has also been demonstrated [91], suggesting that ubiquitination can be a signal to induce selective autophagy. In contrast to the above Pub4-mediated degradation pathway of ubiquitin labelling of single damaged chloroplasts, the selective autophagic clearance of damaged chloroplasts by chlorophagy is caused by membrane potential imbalance [92], which is not related to ubiquitin labelling. Chlorophagy and PUB4-associated ubiquitination independently promote protein turnover to control oxidative damage and nutrient recovery.

### Chloroplast vesiculation pathway

Another degradation pathway under chloroplast stress conditions is CV-containing vesicles (CCVs) [93]. The nuclear-encoded gene CV induces the formation of thylakoid protein-containing vesicles, and CCVs are released from the chloroplast and move into the vacuole for degradation. CV is activated during senescence and abiotic stress, such as salt stress and methyl viologen (MV)-induced oxidative stress. Overexpression of CV leads to premature senescence and chloroplast degradation, whereas silencing CV improves chloroplast stability and prevents abiotic stress-induced senescence [93]. CCV is independent of the autophagosome-associated protein ATG8 and forms in the autophagic mutant ATG5, suggesting that CCV formation is independent of the autophagic pathway. Therefore, CCV is a novel pathway independent of autophagy that selectively removes chloroplast components for vacuolar degradation.

## Conclusions and Prospects

Environmental constraints, including abiotic stressors such as salt, drought, cold, and extreme temperatures, severely limit crop productivity. Chloroplasts have received increasing attention as important regulators of the plant stress response. Current developments in high-throughput expression profiling, chloroplast proteomics, gene editing technology, and other genetic methods can help researchers investigate the stress tolerance mechanism network involved in chloroplasts, such as the TOC-TIC transport apparatus, chloroplast proteome remodelling, operational signal sensing, transduction, and downstream regulatory factors. New findings on the chloroplast and other plastid pathways involved in abiotic stress response can provide new ideas for future plant stress resistance studies.

The import and selective degradation of chloroplast proteins are key processes regulating chloroplast quality and are essential for maintaining chloroplast homeostasis and normal function under environmental stress. As a protein import apparatus, the TOC-TIC system strictly monitors the protein import flux under stress conditions, reduces the photooxidative damage caused by photosynthetic protein import, and increases the import of nonphotosynthetic proteins related to ROS scavenging and stress tolerance. The study by Dutta *et al*. [26] showed that Toc159/Toc33 in *Arabidopsis* focused on the import of photosynthetic proteins, whereas Toc132 or Toc120/Toc34 focused on the import of nonphotosynthetic proteins. Our study also found that the tomato Toc159 receptor family is responsible for transporting different types of precursor proteins [94]. In future studies, the functions of the TOC-TIC apparatus and specific transport substrates and the promotion of organelle homeostasis through dynamic regulation of the chloroplast proteome will be emphasized. The current research on the mechanism of TOC-TIC import is only the beginning of a comprehensive understanding of chloroplast protein turnover.

The main function of plastid reverse signaling is to regulate the expression of nuclear-encoded chloroplast-related genes, maintain chloroplast balance, and support the normal operation of photosynthesis. Therefore, managing plastid reverse signaling under environmental stress is an important strategy for targeting and controlling chloroplast quality. Significant research progress has been made on retrograde signaling in the chloroplast since its first introduction in 1979. However, due to the complexity of chloroplast signaling, our understanding of these signals remains superficial, especially in terms of triggering other key hormonal signaling pathways involved in stress adaptation. Although the chloroplast is an important site for the biosynthesis of plant stress hormones, it is unknown how the chloroplast retrograde signaling pathway interacts with the salicylic acid (SA), jasmonic acid (JA), and abscisic acid (ABA) signaling pathways.

Diverse and complex protein turnover systems and chloroplast signaling could allow plants to optimally coordinate chloroplast turnover, ensuring health throughout the life cycle and overcoming environmental stress. In-depth studies of chloroplasts in stress responses have significantly contributed to the understanding of the complexity of plant stress response networks. In the future, molecular breeding and genetic engineering methods will be adopted to improve the yield stability of crop plants under environmental stress and to impart high-quality traits to crop products. As food and fuel demands increase and arable land decreases, chloroplast quality regulation will be critical to our future survival.

## Supporting information

429Table1
