# Supplementary material for: Research progress on maintaining chloroplast homeostasis under stress conditions: a review: Chloroplast homeostasis under stress conditions
Source: Acta Biochim Biophys Sin (Shanghai). 2023 Feb 24;55(2):173–82. doi: 10.3724/abbs.2023022 (PMC10157539; doi:10.3724/abbs.2023022)
Supplement: 429Table1 [file 429Table1.pdf]

Table 1. Source of typical retrograde signals in plants

Group	Typical member	Source	Ref.
ROS	$^1O_2$	Photooxidative damage	[49]
	H_2O_2	products	[50,51]
	$O_2^{\cdot -}$		[52,53]
Tetrapyrrole	Mg-ProtoIX	Chlorophyll biosynthetic intermediates	[54]
	heme	The heme branch of the plastid tetrapyrrole biosynthesis pathway	[55]
	bilins	heme-derived linear tetrapyrroles	[56]
β-Carotene	β-cyclocitral	Carotenoid oxidation products	[44]
Sulfation	PAP	Product of PAPS sulfation reaction catalyzed by cytoplasmic sulfotransferase	[45]
Methylerythritol isoprenoid	MEcPP	A precursor of isoprenoids produced by the plastidial methylerythritol phosphate (MEP) pathway	[46]
Transcription factors	AP2	The family of Apetala 2 (AP2)/ethylene response element binding protein (EREBP) transcription factors	[47]
	Whirly1	Single-stranded DNA-binding protein family member	[57,58]
	ABI4	Master AP2-type transcription factor	[59]
Kinases	MAPK6	Mitogen-activated protein kinase	[48]
